# Single-Cell Transcriptomic Analysis Revealed a Critical Role of SPP1/CD44-Mediated Crosstalk Between Macrophages and Cancer Cells in Glioma

**DOI:** 10.3389/fcell.2021.779319

**Published:** 2021-11-05

**Authors:** Cong He, Luoyan Sheng, Deshen Pan, Shuai Jiang, Li Ding, Xiaojun Ma, Yaohua Liu, Deshui Jia

**Affiliations:** ^1^ Laboratory of Cancer Genomics and Biology, Department of Urology, and Institute of Translational Medicine, Shanghai General Hospital, Shanghai Jiao Tong University School of Medicine, Shanghai, China; ^2^ State Key Laboratory of Genetic Engineering, School of Life Sciences, and Human Phenome Institute, Fudan University, Shanghai, China; ^3^ Department of Neurosurgery, Shanghai General Hospital, Shanghai Jiao Tong University School of Medicine, Shanghai, China; ^4^ Department of Orthopedics, Shanghai General Hospital, Shanghai Jiao Tong University School of Medicine, Shanghai, China

**Keywords:** glioma, single-cell transcriptomic, tumor heterogeneity, SPP1, CD44

## Abstract

High-grade glioma is one of the most lethal human cancers characterized by extensive tumor heterogeneity. In order to identify cellular and molecular mechanisms that drive tumor heterogeneity of this lethal disease, we performed single-cell RNA sequencing analysis of one high-grade glioma. Accordingly, we analyzed the individual cellular components in the ecosystem of this tumor. We found that tumor-associated macrophages are predominant in the immune microenvironment. Furthermore, we identified five distinct subpopulations of tumor cells, including one cycling, two OPC/NPC-like and two MES-like cell subpopulations. Moreover, we revealed the evolutionary transition from the cycling to OPC/NPC-like and MES-like cells by trajectory analysis. Importantly, we found that SPP1/CD44 interaction plays a critical role in macrophage-mediated activation of MES-like cells by exploring the cell-cell communication among all cellular components in the tumor ecosystem. Finally, we showed that high expression levels of both SPP1 and CD44 correlate with an increased infiltration of macrophages and poor prognosis of glioma patients. Taken together, this study provided a single-cell atlas of one high-grade glioma and revealed a critical role of macrophage-mediated SPP1/CD44 signaling in glioma progression, indicating that the SPP1/CD44 axis is a potential target for glioma treatment.

## Introduction

Glioma is the most common type of primary brain cancers (Louis et al., 2016). According to the WHO classification, glioma has been defined as pilocytic astrocytoma (grade I), low-grade astrocytoma (grade II), anaplastic astrocytoma (grade III) and glioblastoma multiforme (GBM, grade IV). Grade III and IV gliomas are regarded as high-grade gliomas with aggressive phenotypes and poor outcomes (Louis et al., 2021). Mutations in the isocitrate dehydrogenase (IDH) genes, including IDH1 and IHD2, have been frequently identified in low-grade glioma, and correlate with favorable prognosis of patients (Agnihotri et al., 2014; Picca et al., 2018). In contrast, glioma with wild-type IDH genes often shows aggressive behavior and poor prognosis (Omuro and DeAngelis, 2013; Picca et al., 2018).

Tumor heterogeneity has been widely described in cancers and has become one of the major reasons that lead to the failure of anti-cancer treatments (Meacham and Morrison, 2013; Dagogo-Jack and Shaw, 2018). High-grade glioma has been shown to exhibit extensive intra-tumoral heterogeneity, which leads to increased resistance to therapy and tumor progression (Bedard et al., 2013; Darmanis et al., 2017). Recently, it has been shown that crosstalk between cellular components in the tumor ecosystem plays important roles in tumor progression. For example, macrophage-derived oncostatin M (OSM) has been revealed to interact with OSMR or LIFR receptors on tumor cells, and induce a mesenchymal-like state transition of GBM cells (Hara et al., 2021). Exploring the diversity and heterogeneity of cellular compositions in the tumor ecosystem not only helps to understand the mechanisms underlying tumor progression, but also provides new directions to develop targeted therapies. However, the intra-tumoral heterogeneity of cellular components and their crosstalk are largely unknown in high-grade glioma. Therefore, there is an urgent need to further dissect the intra-tumoral heterogeneity of glioma.

Recently, single-cell RNA sequencing (scRNA-seq) has been widely employed to dissect the tumor heterogeneity and explore the complexity of the tumor microenvironment (TME) in many cancers (Darmanis et al., 2017; Azizi et al., 2018; Hornburg et al., 2021). Importantly, scRNA-seq has provided unprecedented opportunities to evaluate the regulation, differentiation and interaction of individual cells in the TME (Trapnell et al., 2014; Aibar et al., 2017; Kumar et al., 2018). Moreover, scRNA-seq has been employed to trace cell evolution in the TME (Vento-Tormo et al., 2018). Recently, multi-omics single-cell sequencing techniques have been used to study glioma, especially high-grade glioma (Darmanis et al., 2017; Filbin et al., 2018; Johnston et al., 2019; Zhao et al., 2019; Castellan et al., 2021). For example, Filbin et al analyzed the developmental and oncogenic programs in H3K27M gliomas by scRNA-seq (Filbin et al., 2018). Besides, Zhao et al performed scRNA-seq analysis of a chromosomally unstable GBM cancer stem cell (CSC) line and revealed the impact of chromosomal instability on GBM progression (Zhao et al., 2019). Additionally, YAP/TAZ has been revealed as regulators of stemness and cell plasticity in IDH wild-type GBM by scRNA-seq (Castellan et al., 2021).

In order to further understand the cellular compositions and their functional roles in high-grade glioma, here we performed scRNA-seq analysis of one high-grade glioma with wild-type IDH genes. We provided a single-cell atlas of the cellular components in this tumor, and revealed essential roles of tumor-immune cell interactions in promoting glioma progression, such as the crosstalk between macrophages and tumor cells mediated by SPP1/CD44 interaction. Altogether, this study revealed a critical role of macrophage-mediated SPP1/CD44 signaling in high-grade glioma, providing a new therapeutic target for the precision treatment of glioma.

## Results

### A Single-Cell Atlas of Cellular Components in the Tumor Ecosystem of One Glioma

**FIGURE 1:**
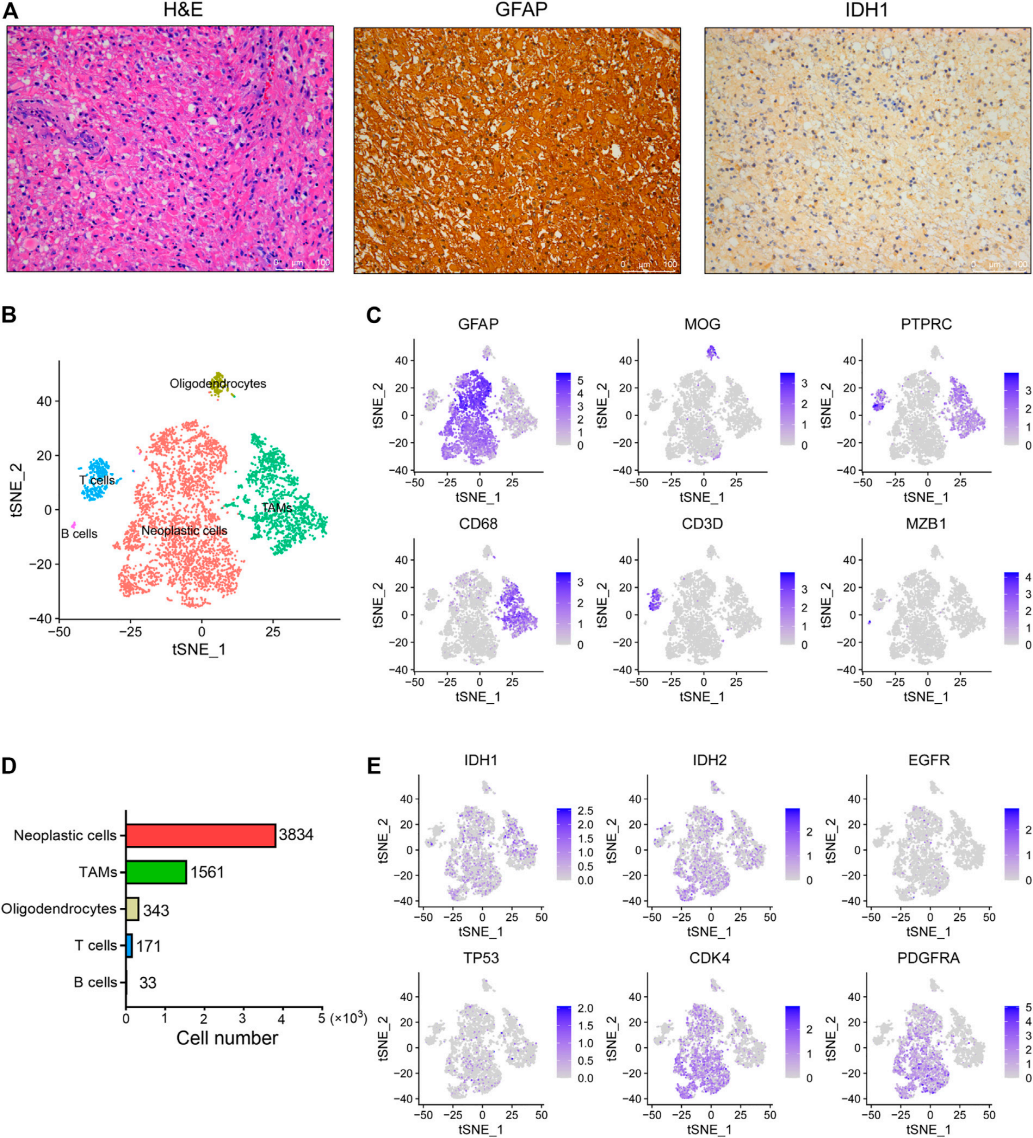
The single-cell landscape of an IDH wild-type glioma. **(A)** Representative images of H&E staining and IHC staining for GFAP and IDH1 proteins in the glioma tissue. Scale bars, 100 μM. **(B)** The t-SNE plot showing 5,945 single cells colored by cell types. TAMs, tumor associated macrophages. **(C)** The t-SNE plot showing the GFAP, MOG, PTPRC, CD68, CD3D and MZB1 expression levels in individual cells. **(D)** The cell number of each cell type in this glioma was displayed. **(E)** The t-SNE plot showing IDH1, IDH2, EGFR, TP53, CDK4 and PDGFRA expression levels in individual cells.

IDH wild-type glioma has been observed with aggressive phenotypes and poor prognosis compared to the IDH mutant glioma (Agnihotri et al., 2014; Picca et al., 2018). In order to identify the underlying mechanisms that drive the development and progression of IDH wild-type glioma, here we performed scRNA-seq analysis of one surgically resected of glioma tumor without IDH1 and IDH2 mutations. First, we showed that this tumor belongs to a grade III anaplastic astrocytoma based on its histopathological features (Figure 1A). Besides, the immunohistochemical staining results revealed that this tumor shows strong expression of the GFAP, a classic marker of astrocytoma. Moreover, this tumor also exhibited positive staining for the IDH1 (Figure 1A).

By use of the 10x Genomics scRNA-seq platform, a total of 5,942 high quality cells were identified in this tumor with an average of 2,265 genes detected per cell. Overall, five distinct cell clusters could be distinguished (Figure 1B). We observed that most cells are GFAP^+^ astrocytoma cells, as well as relatively low proportions of PTRPC^+^ immune cells and MOG^+^ oligodendrocytes in this tumor (Figure 1C). Moreover, we found that tumor-associated macrophages (TAMs) predominate over T cells and B cells in this tumor (Figure 1D). However, we did not observe other cell types in this tumor, such as the tissue-resident astrocytes, vascular endothelial cells and neurons. In addition, we observed that the expression levels of IDH1, IDH2, EGFR and TP53 are relatively low in tumor cells, whereas the expression levels of CDK4 and PDGFRA are relatively high (Figure 1E).

### Landscape of Infiltrating Immune Cells in Glioma

**FIGURE 2:**
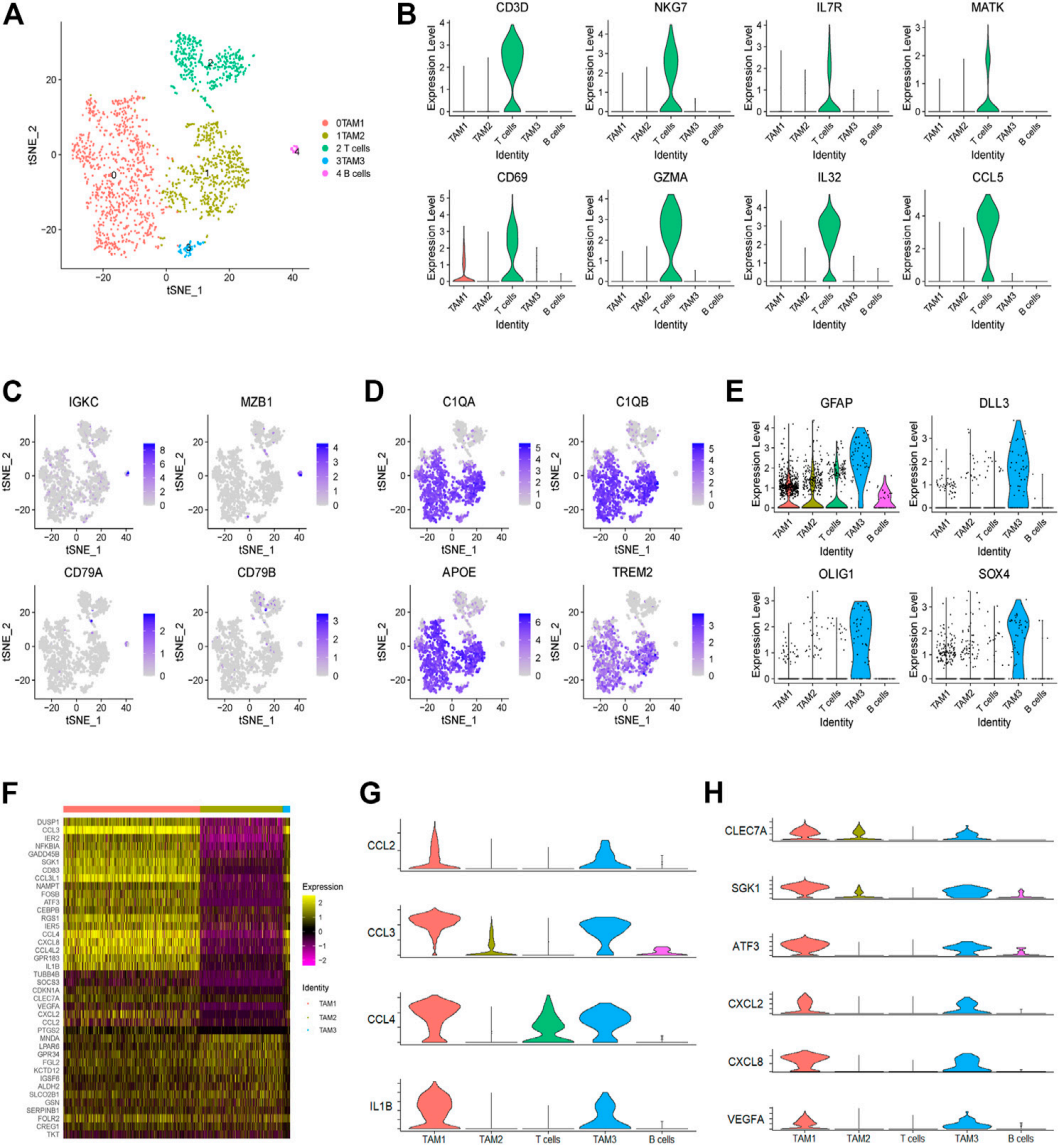
Landscape of infiltrating immune cells in a glioma. **(A)** The t-SNE plot showing 1,765 immune cells colored by cell types. **(B)** The violin plot showing the CD3D, NKG7, IL7R, MATK, CD69, GZMA, IL-32 and CCL5 expression levels in each cluster. **(C)** The t-SNE plot showing the expression levels of B cell markers (IGKC, MZB1, CD79A and CD79B) in individual cells. **(D)** The t-SNE plot showing the expression levels of universal TAM markers of (C1QA, C1QB, APOE and TREM2) in individual cells. **(E)** The violin plot showing the expression levels of tumor cell markers (GFAP, DLL3, OLIG1 and SOX4) in each immune cell cluster. **(F)** Heatmap showing the differential gene expression of three TAM clusters based on the average Log2 (fold change). **(G)** The violin plot showing the expression levels of M1 macrophage-related genes (CCL2, CCL3, CCL4 and IL1B) in each cluster. **(H)** The violin plot showing the expression levels of M2 macrophage-related genes (CLEC7A, SGK1, ATF3, CXCL2, CXCL8 and VEGFA) in each cluster.

In this study, we identified a total of 1,765 infiltrating immune cells in this tumor, which could be further divided into five subpopulations, including three TAM, one T cell and one B cell subpopulations (Figure 2A and Supplementary Figure 1A). Notably, TAMs were observed to be predominant in the TME of this tumor, accounting for over 79% of immune cells. Next, we found that T cells are characterized by high expression of genes related to NKT cells, such as NKG7, IL7R and MATK (Figure 2B and Supplementary Figure 1B). Moreover, we found that B cells show high expression of IGKC and MZB1 genes, but low expression of CD79A and CD79B (Figure 2C). In addition, we observed high expression of universal markers of macrophage (C1QA, C1QB, APOE and TREM2) in all three TAM subpopulations (Figure 2D). Although the three clusters of TAMs have been defined by unsupervised clustering analysis, all the TAMs could be defined as CD45^high^ CX3CR1^+^ CD11b^low^ F4/80^low^ CCR2^−^ macrophages (Supplementary Figure 1C). Interestingly, we found that a subset of TAMs also express genes encoding the markers of glioma cells, such as GFAP, DLL3, OLIG1 and SOX4 (Figure 2E). Consistently, immune cells expressing markers of tumor cells have also been reported in prostate cancer (Chen et al., 2021).

Finally, through comparing gene expression profiling of each TAM subpopulation, we observed that some genes, encoding many cytokines and chemokines, are significantly upregulated in the TAM1 and TAM3 subpopulations, indicating that these TAMs are in an activated state (Figure 2F). For example, we observed high expression of inflammatory molecules (e.g., CCL2, CCL3, CCL4, and IL1B) and M2-like polarization markers (e.g., CLEC7A, SGK1, ATF3, CXCL2, CXCL8, and VEGFA) in the TAM1 and TAM3 cells (Figures 2G,H). Collectively, these results indicated that TAMs in this glioma do not fit into a typical M1 or M2 phenotype, instead they represent a mixed M1/M2-like polarized phenotype.

### Identification of Distinct Cancer Cell Subpopulations in Glioma

**FIGURE 3:**
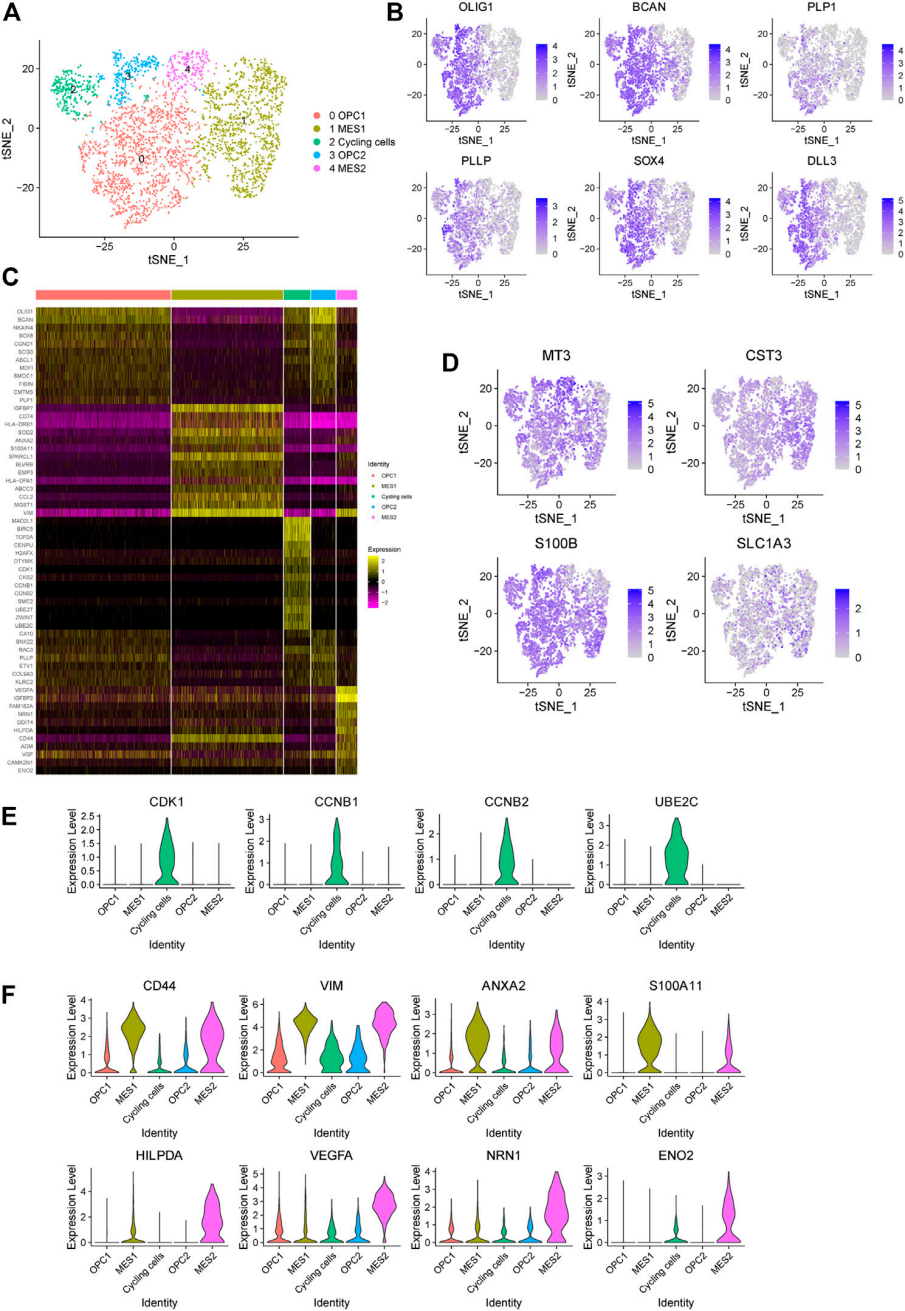
Identification of five cancer cell subpopulations in a glioma. **(A)** The t-SNE plot showing 3,834 cancer cells colored by subtypes. OPC, oligodendrocyte progenitor-like (OPC-like); MES, mesenchymal-like (MES-like). **(B)** The t-SNE plot showing the expression levels of representative markers of OPC-like cells (OLIG1, BCAN, PLP1 and PLLP) and NPC-like cells (SOX4 and DLL3) in individual cells. **(C)** Heatmap displaying the top regulated genes in each cell cluster compared to all other clusters according to the average Log2 (fold change). **(D)** The t-SNE plot showing the expression levels of representative markers of AC-like cells (MT3, CST3, S100B and SLC1A3) in individual cells. **(E)** The violin plot showing the expression levels of representative markers of cycling cells (CDK1, CCNB1, CCNB2 and UBE2C) in each cluster. **(F)** The violin plot showing the expression levels of representative markers of MES1 cells (CD44, VIM, ANXA2 and S100A11) and MES2 cells (HILPDA, VEGFA, NRN1 and ENO2) in each cluster.

In order to dissect the intra-tumoral heterogeneity of cancer cells in this glioma, we further re-clustered all the cancer cells, which also could be divided into five subpopulations (Figure 3A). Previously, high-grade glioma has been identified to be composed of mixed cancer cell populations, including astrocyte-like (AC-like), neural progenitor-like (NPC-like), oligodendrocyte progenitor-like (OPC-like) and mesenchymal-like (MES-like) cells (Phillips et al., 2006; Neftel et al., 2019). Consistently, we found that cells in cluster 0 and three display oligodendroglia lineage markers, such as OLIG1, BCAN, PLP1 and PLLP, which refers to the OPC-like cells (Figures 3B,C). Notably, these cells also show high expression of NPC-like cell signature, including SOX4 and DLL3 (Figure 3B), reflecting the potential of NPC-like toward OPC-like cell differentiation. Here we named these cell clusters as OPC1 and OPC2. Next, we observed that cells in cluster two show high expression of cell cycle-related genes, including CDK1, CCNB1, CCNB2, and UBE2C, which refers to the cycling cell subpopulation (Figure 3E). Interestingly, we observed that these cycling cells also show features of the OPC- and NPC-like cells (Figure 3B).

Next, we found that astrocytic cell markers are widely expressed in all cancer cells, indicating that these cancer cells show features of AC-like cells (Figure 3D). In addition, we observed that cells in cluster one and four exhibit high expression levels of mesenchymal-related genes (e.g., CD44 and VIM), referring to the MES-like cells (Figure 3F). Notably, we found that cells in cluster four shows an increased expression of genes related to the hypoxia (e.g., HILPDA), tumor angiogenesis (e.g., VEGF and NRN1) and glycolytic (e.g., ENO2) (Figure 3F). Therefore, we defined these two MES-like subpopulations as hypoxia-independent (MES1, cluster 1) and hypoxia-dependent (MES2, cluster 4) cells. Together, our findings suggested that this high-grade glioma tumor contains a mixed population of four major cell states.

### Trajectory Analysis of Cancer Cells in Glioma

**FIGURE 4:**
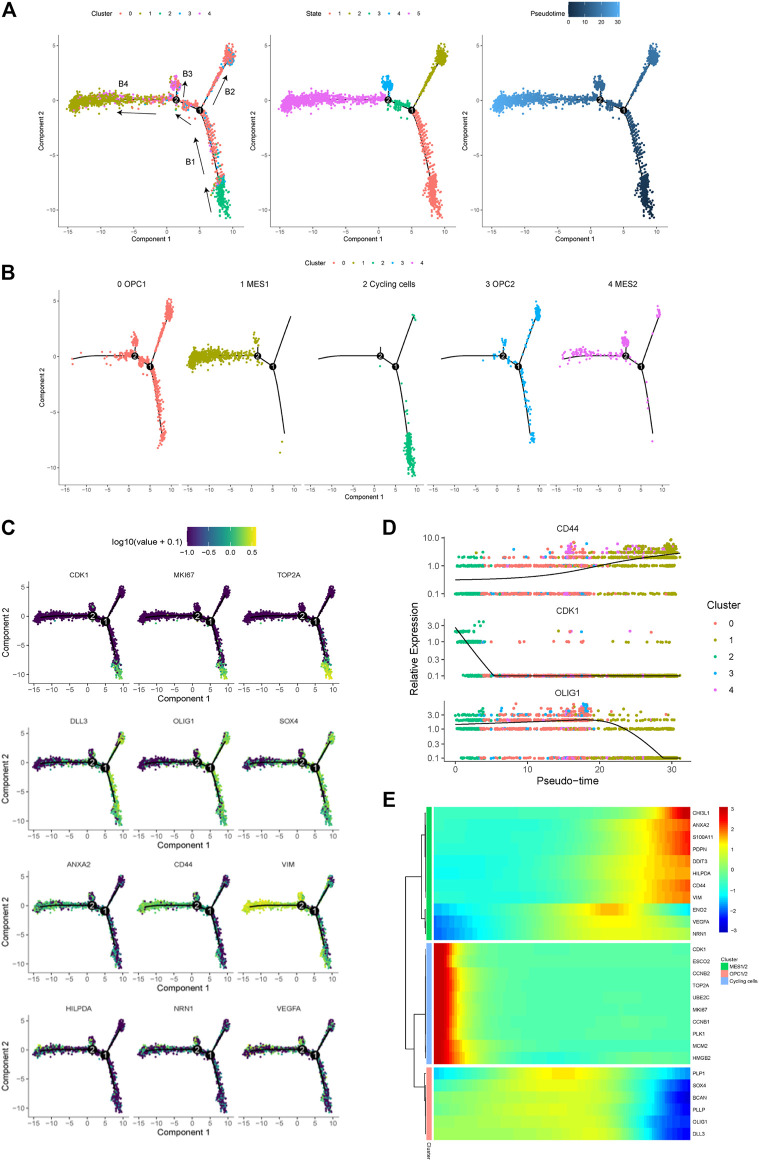
Trajectory analysis of cancer cells in a glioma. **(A)** Pseudotime trajectory analysis revealed four branches (B1, B2, B3 and B4) of cancer cell evolution in this glioma. Cancer cells plotted on the trajectory were colored by clusters (left), states (middle) and pseudotime (right). Black arrows indicated the direction of evolution. **(B)** The location of five cancer cell subpopulations projected on the trajectory. **(C)** The expression of representative markers of cycling cells (CDK1, MKI67 and TOP2A), OPC/NPC-like cells (DLL3, OLIG1 and SOX4), and MES-like cells (ANXA2, CD44, VIM, HILPDA, NRN1 and VEGFA) plotted on the trajectory. **(D)** The relative expression levels of the CD44, CDK1 and OLIG1 genes spanning cycling, OPC/NPC-like to MES-like cells over pseudotime. **(E)** Heatmap of the top upregulated genes in cycling, OPC/NPC-like and MES-like cells over pseudotime.

In order to trace the dynamic evolution of glioma cells during tumor progression, we performed trajectory analysis of the cancer cells. Overall, we identified four branches (represented as “B1,” “B2,” “B3,” and “B4”) and four states of cancer cells (Figure 4A). We found that the trajectory’s root (B1) is composed by majority of the cycling cells (cluster 2) and some OPC- and NPC-like cells. Moreover, we observed that the B3 and B4 branches are composed of most cells from the MES-like cells (cluster one and 4), indicating that cells in these branches are derived from cells in B1 branch (Figure 4B). Therefore, we could postulate that glioma cells evolve from B1 through branch point 1, which further differentiate into two branches, the B2 (OPC- and NPC-like cells) and the B4 (MES-like cells). Therefore, these results indicated the malignant progression of glioma cells, spanning from the cycling, OPC/NPC-like, eventually to MES-like cells. Furthermore, we displayed the expression levels of representative genes in distinct cell states along the trajectory tree (Figure 4C), and also the expression of representative markers of cycling, OPC/NPC-like and MES-like cells over pseudo-time (Figure 4D). In addition, the differentially expressed genes in cycling, OPC/NPC-like and MES-like cells were plotted (Figure 4E). Together, these findings indicated that a dynamic evolution of cancer cells drives the intra-tumoral heterogeneity of glioma.

### Interrogation of Cell-Cell Interactions Between Cellular Components in Glioma

**FIGURE 5:**
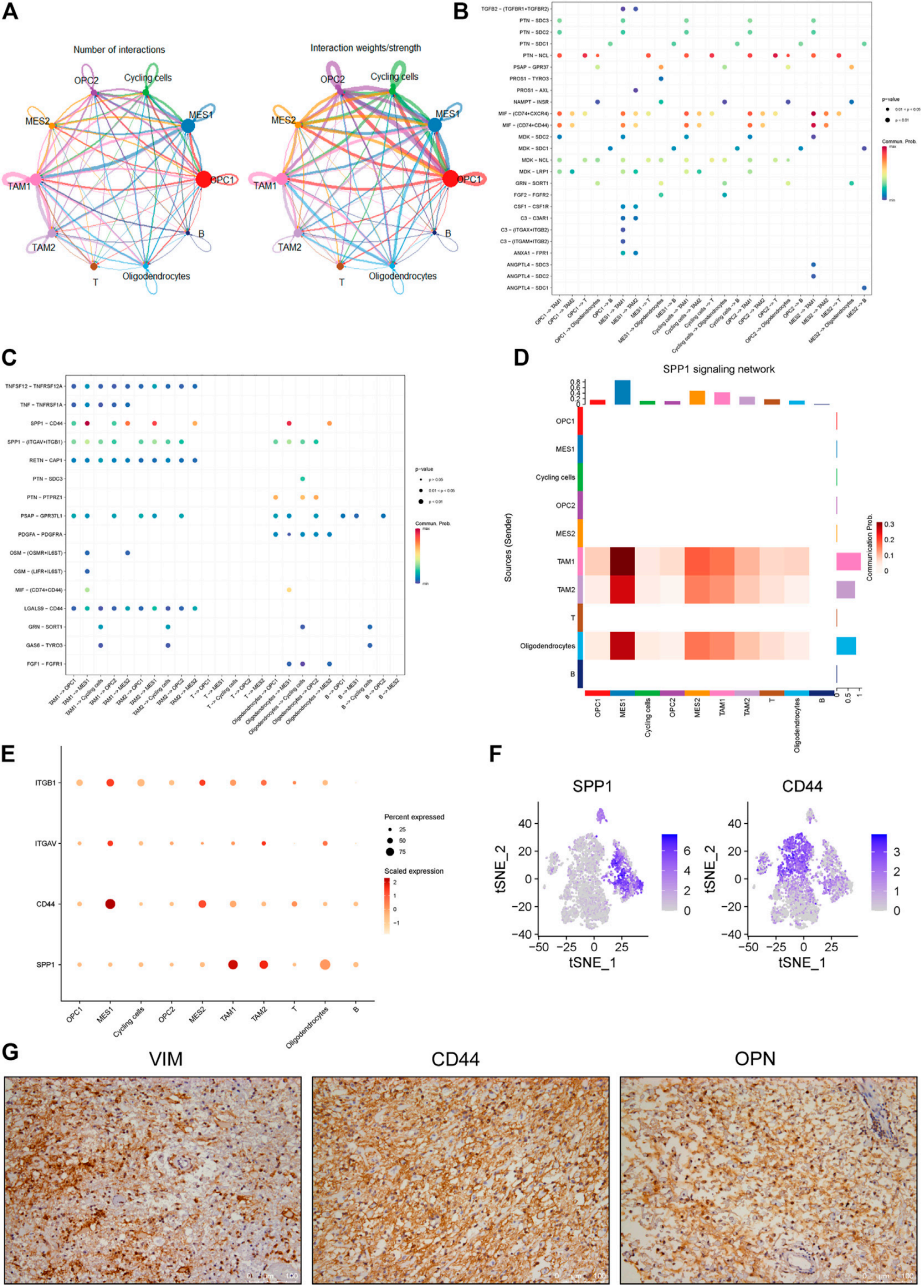
Identification of cell-cell interactions in a glioma ecosystem. **(A)** Circle plots displaying the number (left) and weights (right) of ligand-receptor interactions between distinct cellular components, respectively. Clusters are distinguished by colors. The circle size is proportional to the number of cells in each cluster, the edge width represents communication probability. **(B-C)** Dot plots showing the effects of tumor cells on stromal cells **(B)** and stromal cells on tumor cells **(C)**, respectively. Dot colors refer to communication probabilities whereas dot sizes represent the calculated *p* values. Empty space indicates that the communication probability is zero. The *p* value was calculated by one-sided permutation test. **(D)** Heatmap showing the SPP1-related signaling networks. *Y* axis refers to cells that send the signal, whereas *x* axis refers to cells that receive the signal. The color bar represents the communication probability. The columns on the upper and right sides refer to the accumulation of the signal intensity of the *y* and *x* axis, respectively. **(E)** The dot plot showing the expression levels of representative genes (ITGB1, ITGAV, CD44 and SPP1) in the SPP1 signaling network. The color bar represents the signal intensity. **(F)** The t-SNE plot showing the SPP1 and CD44 expression levels in all cells of this glioma. **(G)** The representative images of IHC staining for VIMENTIN, CD44 and OPN proteins in the glioma tumor. Scale bars, 100 μM.

In order to identify mechanisms that drive the evolution of glioma cells, we investigated the cell-cell communication mediated by ligand-receptor interactions across all cell components. Accordingly, a total of 512 pairs of interactions were identified in this glioma (Supplementary Table 1). We observed that all cell subpopulations are connected with each other (Figure 5A and Supplementary Figure 2A). First, we focused on exploring the relationship between tumor and stromal cells. Many interactions between cancer cells and other cell types in TME were observed, especially the TAM and T cells (Figure 5B). For example, we found that all cancer cell subpopulations express macrophage migration inhibitory factor (MIF), which could bind to CD74 receptor on the TAM and T cells (Figure 5B and Supplementary Figure 2B, C). Besides, we found increased expression of angiogenic CXC chemokines in the TAM1 macrophages, including CXCL2 and CXCL8 (Supplementary Figure 2C, D). Notably, MIF has been reported to be overexpressed in several cancers, and the interaction between MIF and CD74 has also been shown to promote cell proliferation and tumor growth (Xu et al., 2008; McClelland et al., 2009; Cheng et al., 2011; Zheng et al., 2012).

Furthermore, we investigated the roles of stromal cells, especially the TAMs, in regulating cancer cells. We observed multiple ligands and receptors interactions related to tumor progression. For example, we observed the interaction between growth arrest-specific 6 (GAS6) expressed by TAMs and its cognate receptor TYRO3 expressed on the cycling tumor cells (Figure 5C and Supplementary Figure 2E), which was reported to positively correlate with tumor growth (Wu et al., 2018). Also, we observed OSM/OSMR and OSM/LIFR interaction pairs between the TAMs and MES-like cells (Figure 5C and Supplementary Figure 2F), which was consistent with previous findings in glioma (Hara et al., 2021). Notably, we found that SPP1/CD44 interaction pair stands out among all interaction pairs, which primarily mediates the crosstalk between the TAMs and MES-like cells and shows the highest score (Figures 5C–E). OPN encoded by the SPP1 gene has been shown to play critical roles in tumor progression by interacting with different receptors, such as integrin and CD44 receptors (Rangaswami et al., 2006). Here we found that the SPP1 gene mainly expresses in the macrophages rather than tumor cells, whereas the CD44 primarily expresses in the MES-like cells (Figure 5F), suggesting that TAM-secreted OPN may regulate the mesenchymal phenotype of glioma by interacting with CD44 on tumor cells. Furthermore, we confirmed the strong expression of CD44, OPN and VIMENTIN in this glioma by immunohistochemical analysis (Figure 5G). Collectively, these findings indicated that the macrophage-mediated SPP1/CD44 interaction might contribute to the induction of MES-like glioma cells.

### High CD44 and SPP1 Expression Correlated With Poor Prognosis of Glioma Patients

**FIGURE 6:**
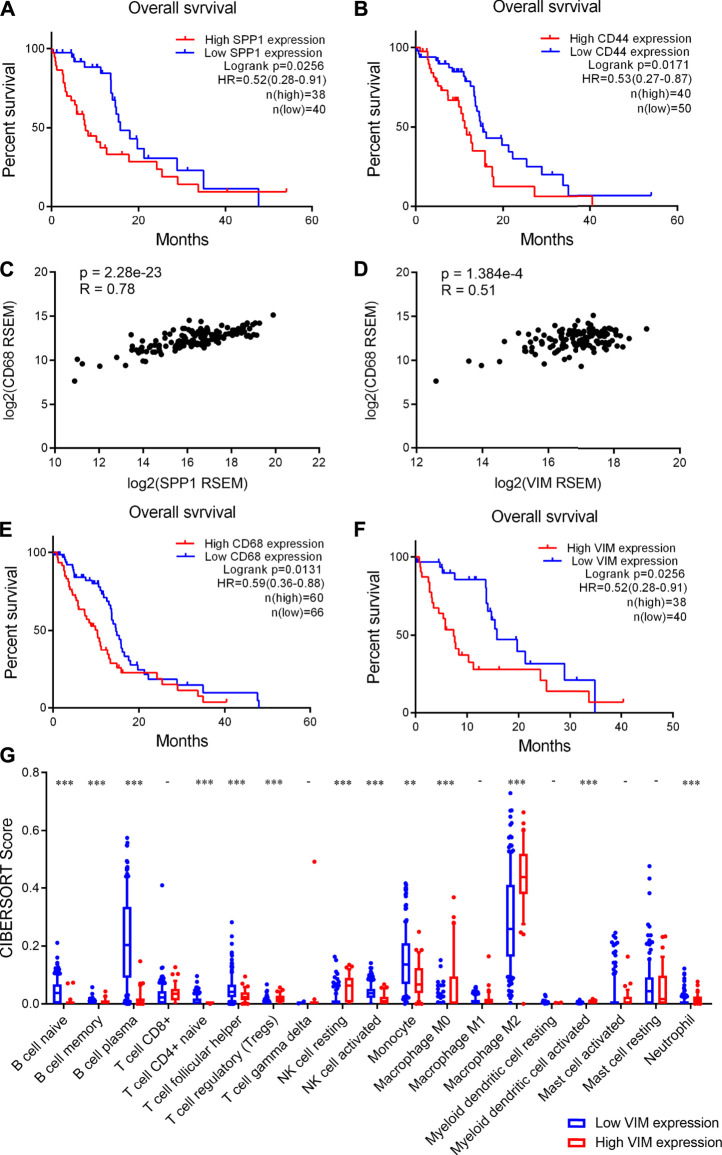
High CD44 and SPP1 expression correlated with increased infiltration of macrophages and poor prognosis of glioma patients. **(A-B)** High SPP1 and CD44 expression correlated with poor prognosis of glioma patients respectively. Log-rank test, *p* < 0.05 was considered significant. **(C-D)** Positive correlations between CD68 and SPP1 or VIM expression levels in human glioma tumors. *p* < 0.05 was considered significant. **(E-F)** High CD68 and VIM expression correlated with an unfavorable prognosis of glioma patients. Log-rank test, *p* < 0.05 was considered significant. **(G)** Box plot showing the scores of infiltrated immune cell subtypes between glioma tumors with low and high VIM expression. The *x* axis represents the 19 subtypes of infiltrated immune cells, the *y* axis represents the scores of infiltrated immune cells. Wilcoxon’s test, **, *p* < 0.01; ***, *p* < 0.001.

Finally, we investigated the clinical significance of increased expression of SPP1 and CD44 in glioma. We found that high SPP1 expression correlate with a shorter overall survival time of glioma patients (Figure 6A). Similarly, high CD44 expression was observed to correlate with decreased overall survival time of glioma patients (Figure 6B). Moreover, we showed that there is a strongly positive correlation between SPP1 and CD68 expression levels in glioma samples (Figure 6C), Similarly, positive correlation between VIM and CD68 expression levels in glioma samples was observed (Figure 6D). Importantly, we also observed a significant correlation between CD44 and VIM expression in glioma samples (Supplementary Figure 3). These results suggested that high SPP1 and VIM expression also associates with an increased infiltration of macrophages in glioma. Consistently, we found that high CD68 and VIM expression correlate with poor prognosis of glioma patients (Figures 6E,F). Taken together, these findings suggested that MES-like cells enriched tumors show increased infiltration of macrophages and a worse prognosis.

In addition, we analyzed the infiltration of immune cells in human glioma tissues. Accordingly, we found that the infiltration of B cells is significantly lower in the tumors with high VIM expression than those with low VIM expression (Figure 6G). Moreover, we observed that the infiltration of CD4 naïve T cells and activated NK cells significantly decreases, whereas Treg cells and resting NK cells significantly increases (Figure 6G **)**. Importantly, we found that M2 macrophages are more abundant in the tumors with high VIM expression (Figure 6G). Together, these findings indicated that glioma tumors showing MES-like features are characterized by an immunosuppression microenvironment.

## Discussion

High-grade glioma has been a refractory cancer type with poor prognosis. In order to identify new mechanisms that drive glioma progression, here we explored the cellular compositions of one high-grade glioma by scRNA-seq. We not only revealed the intra-tumoral heterogeneity of this tumor, but also identified several new findings in glioma. First, we found that the TAMs are predominant in this tumor while the proportions of T cells and B cells are relatively low. Although accumulating evidence has indicated that Treg cells widely exist within glioma tissues (Hussain et al., 2006), we did not observe any Foxp3^+^ Treg cells in this tumor. Besides, we found that all the three TAM clusters belong to CD45^high^ CX3CR1^+^ CD11b^low^ F4/80^low^ CCR2^−^ subtype of macrophages, therefore, it was hard to distinguish the infiltration of bone marrow-derived macrophages (BMDM) and CNS-resident microglia, which was consistent with the previous reports (Badie and Schartner, 2000; Müller et al., 2015; Hambardzumyan et al., 2016). Instead, we observed that these TAMs exhibit features of both M1 and M2 phenotypes, which reflects the heterogeneity of TAMs. Interestingly, we found that the TAM1 and TAM3 cells also show high expression levels of multiple oncogenes, such as DUSP1, IER2 and NAMPT, which have been implicated in cancer progression (Neeb et al., 2012; Teng et al., 2018; Zhang et al., 2019). Together, our findings suggested that these TAMs more likely play pro-tumor roles in glioma.

Furthermore, five subpopulations of glioma cells have been identified in this tumor. We revealed the evolution of tumor cells from cycling through OPC/NPC-like to MES-like cells, which further confirmed the aggressive characteristics of the MES-like cells. The cycling cells have been reported to process increased proliferation and differentiation abilities and characteristics of cancer stem cells (Ma et al., 2018; Zhai et al., 2020). Here we found that the cycling cells are the origins of OPC/NPC-like and MES-like cells in glioma, indicating that these cycling cells process the property of cancer stem cells.

In addition, we found that the SPP1/CD44 signaling plays important roles in the induction of MES-like tumor cells. Notably, the SPP1/CD44 signaling in the perivascular niche has been shown to promote the stem cell-like properties and radiation resistance of glioma cells (Pietras et al., 2014). Moreover, the increased SPP1 expression has been shown to induce GBM-associated macrophage infiltration and associate with the poor prognosis of GBM patients (Wei et al., 2019). Similarly, high CD44 expression has also been reported to drive glioma progression and correlate with poor prognosis of GBM patients (Si et al., 2020). However, the macrophage-mediated SPP1/CD44 signaling in driving glioma progression has rarely been reported. Therefore, our findings highlighted the importance of targeting SPP1/CD44-mediated macrophage-tumor cell interaction in anti-glioma treatment.

However, this study has some limitations. First, only one glioma specimen was analyzed by scRNA-seq and the number of cells sequenced was relatively low. Therefore, it was hard to identify rare cell types in glioma. Second, the biological roles of cell-cell communication mediated by ligand-receptor pairs, including the SPP1/CD44 pair, have not been determined by functional experiments, which need to be accomplished in future studies. Third, although the macrophage-mediated SPP1/CD44 signaling has been implicated in regulating the evolution of MES-like tumor cells in this study, the precise mechanisms driving the transition from OPC/NPC-like to MES-like cells have not been investigated, which need to be addressed in the future.

In conclusion, this study dissected the intra-tumoral heterogeneity of one high-grade glioma, revealed the critical roles of TAMs in glioma progression, providing a rationale to target tumor-immune cell interactions for personalized glioma therapy.

## Materials and Methods

### Human Specimen

The glioma specimen was collected from a patient who received surgery in Shanghai General Hospital. The patient did not receive any treatment before the surgery and provided informed consent. This study was performed under the protocol (2019SQ175) approved by the Institutional Review Board at Shanghai General Hospital and in accordance with ethical guidelines (Declaration of Helsinki). The fresh tumor specimen was processed for scRNA-seq analysis in 4 h after resection. The histology of this tumor was confirmed by qualified pathologists.

### Single-Cell Suspension Preparation

Fresh tissue was transported in cold PBS on ice. Tissue was washed three times with PBS to remove blood, then cut into tiny pieces (∼1–2 mm^3^) and enzymatically dissociated by 1 mg/ml Type IV collagenase for 45 min at 37°C. The dissociation process was carried out in a shaking incubator with. Undigested tissue masses were removed using 70-μM strainers (Corning), and red blood cells (RBC) were removed by use of RBC lysis buffer (Biolegend). The dissociated cells were washed twice with PBS containing 0.04% bovine serum albumin (BSA, Sigma-Aldrich). Finally, cell viability was examined by Trypan blue (Invitrogen) staining.

### scRNA Sequencing

In this study, 7,000 cells were loaded to generate the library. The library was constructed using 10X Genomics Chromium Single Cell 3′ kit (V2 chemistry) following the protocol provided by the manufacturer. The library was sequenced on the Illumina HiSeq platform using paired end 150 bp mode.

### scRNA-Seq Data Processing

The raw sequencing reads were mapped to the human reference genome (GRCh38) using the CellRanger (version 4.0.0) with default parameters. The Gene-Barcode matrices were generated by calculating unique molecular identifiers (UMIs) and filtering non-cell-related barcodes, which contain the barcoded cells and gene expression counts. The output was then imported into Seurat (version 4.0.3) R toolkit for quality control (QC) and downstream function analysis (Zhou and Jin, 2020). Unless otherwise specified, all functions were run with default parameters. In particular, low quality cells (< 200 genes per cell, < 3 cells per gene and > 10% mitochondrial genes) were excluded. We used the *LogNormalize* function to normalize the data, which normalized the feature expression level through the total expression of each cell. We applied the *FindVariableFeatures* function to count highly variable features and returned the selected number of features (2000 by default). Principal component analysis (PCA) was used to reduce dimension, followed by clustering analysis in PCA space using a graph-based clustering method. t-SNE was used to visualize the identified clusters. Finally, the Seurat functions, including *DotPlot, VlnPlot, FeaturePlot* and *Heatmap*, were used to visualize the gene expression, and the *FindAllMarkers* function was employed to identify markers for a specific cluster against all remaining clusters.

### Histological and Immunohistochemical Staining

The glioma tissue was fixed in 10% neutral buffered formalin (NBF) and embedded in paraffin. 5 µM thick sections were prepared and subjected to hematoxylin and eosin (H&E) and immunohistochemical (IHC) staining using standard protocols. The following primary antibodies were used: anti-GFAP (Abcam, ab68428), anti-IDH1 (Abcam, ab256557), anti-Vimentin (Cell Signaling Technology, 5,741), anti-CD44 (Abcam, ab157107), and anti-OPN (Abcam, ab214050). Peroxidase AffiniPure Goat anti-rabbit lgG secondary antibody (Jackson ImmunoResearch, 111-035-003) was used. Images were acquired using Leica DM6 B microscope.

### Trajectory Analysis

Pseudotime trajectory analysis was performed using Monocle (version 2.20.0). Briefly, a group of ordering genes with differential expression among dictinct tumor cell clusters were selected through differentialGeneTest Function and filtered by *q* value < 0.01 and *p* value < 0.01. Then cells were projected onto the space and arranged into a trajectory with branch points. Cells in the same segment of the trajectory were grouped into the same state.

### Cell-Cell Communication Analysis

The CellChat (version 1.1.0) R package was used to infer, analyze and visualize cell-cell communication among cellular components in the glioma ecosystem. The Seurat normalized expression matrix was used for ligand-receptor interaction analysis. A known list of ligand-receptor pairs was obtained from CellChatDB, a database of literature-supported ligands and receptors interactions in both mouse and human. We first identified overexpressed ligands or receptors among cell types. The gene expression data were then projected onto the protein-protein interaction (PPI) network. If the ligands or receptors overexpressed, the interactions between the overexpressed ligands and receptors were recognized. The communication probability was inferred by calculating the communication probability of all ligands and receptors interactions associated with each signal pathway. The circle plot, bubble diagram or heatmap was used to visualize cellular interaction of specific signal pathway or ligand-receptor.

### Survival Analysis

The cBioportal database (http://www.cbioportal.org/public-portal/) was used to access gene expression and survival information of glioma patients (Merged low-grade gliomas and GBM, TCGA, mRNA Expression (RNA seq V2 RSEM)) (McLendon et al., 2008; Brennan et al., 2013; Ceccarelli et al., 2016). First, the median value of the SPP1, CD44, CD68 and VIM genes in all tumors were calculated. Based on the gene expression value of each gene in individual tumor, tumors were allocated into high expression group and low expression group. Kaplan-Meier estimator was used to estimate the overall survival time of patients in each group. Statistical significances between the high and low expression group were calculated by Log-Rank (Mantel-Cox) test using GraphPad Prism 5.

### Evaluation of Infiltrated Immune Cells

In order to determine the infiltration of immune cells in human glioma tissues, the bulk RNA-seq data of human gliomas from the TCGA database was used. The Cell-type Identification By Estimating Relative Subsets Of RNA Transcripts (CIBERSORT) algorithm was employed to infer the proportion of infiltrated immune cells as described previously (Newman et al., 2015). The quality of tumor samples was first examined and only samples with *p* value <0.05 were selected for next analysis. The scores of infiltrated immune cells in each sample were visualized by boxplot.

## Data Availability

The original contributions presented in the study are publicly available. This data can be found here: National Center for Biotechnology Information (NCBI) BioProject database under accession number GSE185231.
